# Satisfaction of well-controlled type 2 diabetes patients with three-monthly and six-monthly monitoring

**DOI:** 10.1186/1471-2296-14-107

**Published:** 2013-07-30

**Authors:** Paulien R Wermeling, Jolien Janssen, Kees J Gorter, Joline WJ Beulens, Guy EHM Rutten

**Affiliations:** 1 Julius Center for Health Sciences and Primary Care, University Medical Center, Utrecht, Netherlands

## Abstract

**Background:**

Patient’s satisfaction with monitoring frequency is of interest when implementing six-monthly monitoring for well-controlled type 2 diabetes patients. Here we want to determine the satisfaction of well-controlled type 2 diabetes patients with either three-monthly or six-monthly diabetes monitoring and their future preference.

**Methods:**

Survey among 2215 well-controlled type 2 diabetes patients (not using insulin, HbA1c ≤58 mmol/mol, systolic blood pressure ≤145 mmHg and total cholesterol ≤5.2 mmol/l) who participated in the EFFIMODI study, a randomised controlled patient-preference equivalence trial. At baseline, participants were asked whether they had a strong preference for three-monthly or six-monthly monitoring or not. If not, they were randomised to either three-monthly or six-monthly monitoring, while the others were monitored according to their preference. After eighteen months, all participants were asked whether they were satisfied with the monitoring frequency and about their future preference. Patient characteristics associated with satisfaction were also examined.

**Results:**

Most patients (70.8%) would like to continue their monitoring frequency. Patients from the preference groups were more often satisfied than randomised patients (92.7% and 88.1%, respectively) and patients monitored three-monthly were more often satisfied than patients monitored six-monthly (93.5% and 88.5%, respectively). Higher age, better physical health, less diabetes-related distress, higher diabetes treatment satisfaction and less perceived hyper- and hypoglycaemias were associated with a higher monitoring satisfaction.

**Conclusions:**

Most well-controlled type 2 diabetes patients were satisfied with their monitoring frequency and would like to continue it. Although the satisfaction for three-monthly monitoring was slightly higher, the satisfaction with six-monthly monitoring was still rather high (88.5%).

**Trial registration:**

Current controlled trials ISRCTN93201802.

## Background

Worldwide, the number of people with type 2 diabetes was 366 million in 2011 and this number is likely to increase with 50% by 2030 [1]. International diabetes guidelines recommend different monitoring frequencies ranging from one to four times a year, but these guidelines are not evidence-based [2,3]. Here monitoring refers to the role of the diabetes care provider who regularly checks the health status of the patient, the cardiometabolic targets included.

If part of the patients with diabetes would visit their general practice less frequently, this could lessen the patients’ and health care providers’ diabetes-related burden and provide savings in healthcare costs. We could demonstrate that six-monthly monitoring of well-controlled diabetes patients who had no strong preference with regard to their diabetes monitoring frequency in primary care achieved comparable cardiometabolic control compared to three-monthly monitoring (submitted).

However, two out of three patients had a strong preference with regard to the monitoring frequency [4]. These preferences might hamper the implementation of six-monthly monitoring. Patients who receive their preferred treatment may have better compliance and be more motivated to follow treatment regimens [5,6].

Besides costs savings, the satisfaction of the patients with their monitoring frequency is of interest when implementing six-monthly monitoring, since patient satisfaction is associated with treatment adherence [7,8]. To our knowledge, no research has been performed on satisfaction with monitoring frequency and which factors are associated with it.

Therefore, we aimed to assess patient’s satisfaction with either three-monthly or six-monthly diabetes monitoring after eighteen months of experience in both patients with and without a strong preference at the start of that period. We also determined which patient characteristics and health-related factors are associated with this satisfaction and what patients prefer for the future with regard to their diabetes monitoring.

## Methods

### Study design and patients

This study is part of a randomised controlled patient-preference equivalence trial in primary care. Type 2 diabetes patients were recruited in general practices across the Netherlands from April 2009 to August 2010. Patients were eligible if between 40 and 80 years old, diagnosed with type 2 diabetes for more than a year, treated by their general practitioner, not on insulin treatment and overall well-controlled, defined as having HbA1c ≤58 mmol/mol (≤7.5%), systolic blood pressure ≤145 mmHg and total cholesterol ≤5.2 mmol/l. We have chosen values a little higher than the currently used targets in the Netherlands [9] in order to create a larger target population.

Eligible patients were contacted by mail by their general practitioner with an information letter and an informed consent form. At baseline, patients were asked whether they had a strong preference for three-monthly or six-monthly monitoring. Those with a strong preference for either three-monthly or six-monthly monitoring were treated according to their preference, while those without a strong preference were randomised to either three-monthly or six-monthly monitoring. After returning the informed consent form, all participants received a postal questionnaire to be completed at home and after eighteen months they received the end-of-study questionnaire.

The study was approved by the Medical Research Ethics Committee of the University Medical Center Utrecht (protocol number 08–453). A detailed protocol of the EFFIMODI study has been published elsewhere [10]. In this paper we describe the satisfaction of well-controlled type 2 diabetes patients with three-monthly and six-monthly monitoring.

### Measurement of patient’s satisfaction and preference for the monitoring frequency in the future

In the end-of-study questionnaire, the following two questions were included:

1) How satisfied were you with either three-monthly or six-monthly monitoring? (not at all, not, neutral, moderate, very much)

2) How often would you like to be monitored in the future? (every three months, every six months, no preference/I do not care, every (filled in by the patient) months)

### Measurement of determinants of patient’s satisfaction

Several possible determinants of patient’s satisfaction were assessed. These were derived from the patient questionnaires or from the medical data collected by a case report form filled in by the general practitioner or practice nurse.

Both the baseline and end-of-study questionnaire included information on age, gender, smoking, health status (measured with the SF-36 [11] and EQ VAS [12]), diabetes-related distress (measured with the PAID [13]) and satisfaction with diabetes treatment and experienced hyper- or hypoglycaemias (measured with the DTSQ status [14]).

The case report form consisted of information on the year of diabetes diagnosis and comorbidities at baseline (myocardial infarction, angina pectoris, heart surgery, heart failure, stroke, transient ischemic attack, peripheral arterial disease, COPD, rheumatoid arthritis, osteoarthritis of hip or knee or any other diseases). Furthermore, we used the last known measurement of HbA1c, HDL, LDL and total cholesterol levels as well as baseline and final measurements of blood pressure and weight.

### Statistical analysis

Descriptive statistics were used to describe the satisfaction with the monitoring frequency and preference for future monitoring frequency for each study group. For the future monitoring frequency the answers of the second question were categorised into: frequent (every 1–4 months), infrequent (every ≥ 5 months) and does not care.

Then the answers to the first question were categorised into: (very) satisfied, neutral and not (at all) satisfied. The chi square test was used to examine if satisfaction differed between the three-monthly and six-monthly groups (both with and without a preference) and between the preference and randomised groups (both three-monthly and six-monthly monitoring).

Since the group who chose ‘not (at all) satisfied’ was small, this was combined with ‘neutral’ to compare the differences in patient characteristics and health-related factors between patients who were satisfied and those who were not. We used logistic regression for categorical variables and linear regression for continuous variables to demonstrate the association between satisfaction and patient characteristics and health-related factors. In addition, we added the four study groups as interaction terms to the regression models. If not significant, study group was added to the regression model as confounder. Data were analysed with SPSS version 20.

## Results

Of the 4040 patients who were invited to participate, 2215 (54.8%) agreed. Participants had a mean age of 64 years (SD=9) at the start of the study period, 1311 (59%) were male and the mean duration of diabetes was six years (SD=4). Of all participants, 747 patients (33.7%) preferred three-monthly monitoring, 677 (30.6%) preferred six-monthly monitoring, 394 (17.8%) were randomised to three-monthly and 397 (17.9%) to six-monthly monitoring at baseline (Table 1).

**Table 1:** **Number** (%) **of patients who are satisfied with the monitoring frequency and how often they want to be monitored in the future**, **divided per study group**

	**Preferred three-monthly n=747**	**Preferred six-monthly n=677**	**Randomised to three-monthly n=394**	**Randomised to six-monthly n=397**
**Satisfaction with the monitoring frequency**	**n (%)**	**n (%)**	**n (%)**	**n (%)**
Very satisfied	291 (46.3%)	206 (35.6%)	106 (30.3%)	98 (28.1%)
Satisfied	303 (48.2%)	318 (55.0%)	214 (61.1%)	198 (56.7%)
Neutral	29 (4.6%)	39 (6.7%)	25 (7.1%)	38 (10.9%)
Not satisfied	3 (0.5%)	12 (2.1%)	4 (1.1%)	10 (2.9%)
Not at all satisfied	2 (0.3%)	3 (0.5%)	1 (0.3%)	5 (1.4%)
**Preference for future monitoring**				
Frequent (every 1–4 months)	545 (86.6%)	87 (15.0%)	203 (57.8%)	120 (34.4%)
Infrequent (every ≥5 months)	54 (8.6%)	445 (76.6%)	84 (23.9%)	159 (45.6%)
Does not care	30 (4.8%)	49 (8.4%)	64 (18.2%)	70 (20.1%)

### Satisfaction with the monitoring frequency

In Table 1 the satisfaction with the monitoring frequency per study group is shown. Patients from the preference groups were more often (very) satisfied compared to those who were randomised (92.7% and 88.1%, respectively; p=0.001) and patients from the three-monthly groups were more often (very) satisfied with their monitoring frequency compared to those in the six-monthly groups (93.5% and 88.5%, respectively; p<0.001).

Satisfaction with diabetes treatment (DTSQ) did not differ between the four study groups (ANOVA, p=0.454). The three-monthly preference group had a score of 31.8, the six-monthly preference group of 32.2, the randomised three-monthly group of 31.9 and the six-monthly group of 31.8.

### Preference for the monitoring frequency in the future

Most patients (70.8%) would like to continue their monitoring frequency. 11.1% did not care about their future monitoring frequency and the remainder preferred either a more (10.8%) or less (7.2%) frequent monitoring in the future.

Patients who received their preferred monitoring frequency were more likely to insist on continuing their preferred monitoring frequency in the future, both in the three-monthly group (86.6%) and in the six-monthly group (76.6%) (Table 1). Only 19.1% of the patients who did not have a preference at baseline still did not care about the monitoring frequency in the future after their eighteen months of experience in the trial. The patients who were randomised to either three-monthly or six-monthly monitoring were more likely to prefer the monitoring frequency they were assigned to, although this effect was slightly stronger in the three-monthly monitoring group.

### Patient characteristics associated with satisfaction with the monitoring frequency

Satisfaction with the monitoring frequency was associated with: higher age, a better physical health, less diabetes-related distress, higher diabetes treatment satisfaction, less experienced hyper- and hypoglycaemias and a lower LDL and diastolic blood pressure (Table 2). No interaction by study group was observed in the association between satisfaction and patient characteristics and health-related factors. Besides, adding study group as a confounder to the models did not alter the results. Therefore, we only reported the crude p-values.

**Table 2:** **Satisfaction with the monitoring frequency** (**n**=**1905**)

	**Not satisfied n=171**		**Satisfied n=1734**		
	**n**	**Mean ± SD or n (%)**	**n**	**Mean ± SD or n (%)**	**P-value**
Age at baseline	171	62.5 ± 8.9	1734	64.6 ± 8.6	**0**.**003**
Gender, male	171	111 (64.9%)	1734	1029 (59.3%)	0.156
One or more comorbidities at baseline	171	69 (40.4%)	1734	646 (37.3%)	0.425
Duration of diabetes (in years)	167	5.6 ± 3.3	1695	5.9 ± 3.7	0.283
Current smoker*	165	35 (21.2%)	1672	251 (15.0%)	0.137
SF-36 PCS [scale: 0–100]*	137	44.7 ± 10.4	1414	45.7 ± 10.5	**0**.**027**
SF-36 MCS [scale: 0–100]*	137	51.5 ± 10.5	1414	54.1 ± 8.7	0.407
EQ VAS [scale: 0–100]*	155	71.7 ± 13.8	1510	75.2 ± 14.7	0.100
PAID [scale: 0–80]*	152	10.3 ± 11.8	1539	6.5 ± 9.0	<**0**.**001**
DTSQ [scale: 0–36]*	158	26.8 ± 6.4	1607	32.4 ± 4.1	<**0**.**001**
DTSQ hyper [scale: 0–6]*	166	2.2 ± 1.9	1650	1.3 ± 1.7	<**0**.**001**
DTSQ hypo [scale: 0–6]*	166	1.3 ± 1.6	1652	0.8 ± 1.4	**0**.**001**
HbA1c (mmol/mol)**	160	49.3 ± 6.8	1656	48.2 ± 7.5	0.077
HDL cholesterol (mmol/l)**	159	1.2 ± 0.3	1637	1.2 ± 0.3	0.089
LDL cholesterol (mmol/l)**	159	2.4 ± 0.8	1634	2.3 ± 0.7	**0**.**044**
Total cholesterol (mmol/l)**	159	4.3 ± 0.9	1633	4.2 ± 0.8	0.069
Systolic blood pressure (mmHg)*	130	132.9 ± 15.1	1477	134.0 ± 14.7	0.680
Diastolic blood pressure (mmHg)*	130	78.1 ± 9.9	1474	76.3 ± 9.2	**0**.**025**
BMI (kg/m^2^)*	120	29.1 ± 4.2	1373	29.1 ± 4.8	0.609

* Final measurement, corrected for baseline measurement.

** Last known measurement during follow-up.

*SF-36* Short-Form 36, *PCS* Physical Component Score, *MCS* Mental Component Score, *EQ VAS* EuroQol Visual Analogue Scale, *PAID* Problem Areas In Diabetes, *DTSQ* Diabetes Treatment Satisfaction Questionnaire status.

## Discussion

This study showed that most well-controlled type 2 diabetes patients were satisfied with their monitoring frequency whether self-chosen or allocated by randomisation. Besides, the majority would like to continue this frequency in the future. Patients being monitored three-monthly or according to their preference tended to be more satisfied. On the other hand, most patients assigned to their study group by randomisation wished to continue the assigned monitoring frequency in the future.

Overall, patients were satisfied with their monitoring frequency in our study. Despite this, patients who determined their own monitoring frequency were more satisfied. Therefore, in order to increase patient’s satisfaction, patient’s preferences should be taken into account. However, currently the monitoring frequency is mostly determined by the physician in patients with chronic diseases [15-18]. Patients usually prefer a slightly less frequent monitoring compared to physicians [19] or as determined by guidelines [20]. This leads to the question: to whom should we listen?

A study in haemodialysis care showed that patients with less frequent patient-physician contact were less satisfied with their monitoring frequency, although no difference in satisfaction with overall quality of care was found [21]. A randomised equivalence trial comparing three-monthly and six-monthly monitoring in patients with hypertension in primary care showed no significant differences on satisfaction with care [22], although more patients in the six-monthly group thought that their general practitioner did not take their blood pressure problem seriously enough. In accordance with these studies, we also observed that the prevalence of satisfied patients was slightly higher among those randomised to three-monthly monitoring than those randomised to six-monthly monitoring. However, we have also measured satisfaction with the diabetes treatment, which is more comparable to the score used by Birtwhistle et al. Using this score, we could not detect any significant differences between the four study groups. So based on this, the patients who are monitored six-monthly are satisfied with the overall care, although they indicated to be slightly less satisfied with the monitoring frequency than those randomised to three-monthly monitoring.

Patients who had a lower physical health, a higher diabetes-related distress and perceived more hyper- and hypoglycaemias were less satisfied with the monitoring frequency. We also found significant differences for LDL cholesterol and diastolic blood pressure, but the absolute differences were very small. So it seems that well-controlled type 2 diabetes patients based their dissatisfaction with the monitoring frequency on logical reasons. This is in concordance with the preferences of the patients at baseline, where we showed that patients seem to make logical choices regarding the monitoring frequency [4]. These results confirm that patients themselves should also be involved in determining the monitoring frequency. One out of three had a strong preference for three-monthly monitoring and therefore, this should be taken into account.

A major advantage is that treating patients according their preference might improve treatment adherence and thus clinical outcomes [23]. Especially in diabetes care this is of utmost importance, since patients have to deal with treatment adherence and self-management to keep their diabetes under control. This could be achieved by discussing the preferred monitoring frequency with the patient on a structured annual basis. Besides, general practitioners might discuss with the patient their satisfaction with the monitoring frequency.

One of the strengths of this study is that we included a large number of type 2 diabetes patients. Furthermore, extensive patient data were available providing the opportunity to explore both patient characteristics and health-related factors in relation to patient satisfaction. However, it is unknown whether additional patient factors may influence the satisfaction with the monitoring frequency. Unfortunately, we were unable to take unknown variables (such as travel time to the general practice, mean time in the waiting room, mean duration of the visits, compliance to therapy, knowledge of type 2 diabetes and patients’ coping strategies) into account. Another limitation is that some patients might have misunderstood the question on the monitoring frequency. Some patients may have indicated how satisfied they were with the content of the visits instead of with the number of visits. This would also explain some ‘implausible’ answers. For example, 261 patients (13.7%) who liked their monitoring frequency preferred another frequency in the future and 17 patients (0.9%) who disliked their monitoring frequency chose the same frequency in the future (data not shown). However, we believe that because of the low percentage of these inconsistencies, this will not have influenced the direction of our results. Another limitation is that we included only well-controlled patients. However, well-controlled patients are the target population for a looser monitoring interval (every six months) as opposed to more "problematic" diabetes patients, who may require more frequent visits.

Of course diabetes monitoring regards more parameters than HbA1c, blood pressure or cholesterol such as kidney function, micro-albuminuria, micro- and macrovascular complications and health related quality of life. We could demonstrate that in our study population comorbidities were strongly related to health status [24]. These aspects should be monitored in all patients during a longer annual check-up. Obviously the presence or deterioration of any of these parameters may justify a more intensive monitoring frequency.

## Conclusions

Based on the results we conclude that it is feasible to implement six-monthly monitoring in well-controlled type 2 diabetes patients. We have shown that patient’s preferences should be taken into account to increase patient’s satisfaction. Although the satisfaction for three-monthly monitoring was higher than in six-monthly monitoring, the satisfaction with six-monthly monitoring was still rather high (88.5%). We expect that at least two out of three well-controlled type 2 diabetes patients can be eligible for six-monthly monitoring.

## Competing interests

The authors declare that they have no competing interests.

## Authors' contributions

PW, KG and GR participated in the design of the study. PW collected the data. PW and JJ performed the statistical analysis and drafted the manuscript. JB contributed to the analysis and results. JB, KG and GR all reviewed the manuscript and were involved in its critical revision before submission. All authors read and approved the final manuscript.

## Pre-publication history

The pre-publication history for this paper can be accessed here:

http://www.biomedcentral.com/1471-2296/14/107/prepub
